# The anti-HBV effect mediated by a novel recombinant eukaryotic expression vector for IFN-α

**DOI:** 10.1186/1743-422X-10-270

**Published:** 2013-08-29

**Authors:** Haotian Yu, Zhaohua Hou, Qiuju Han, Cai Zhang, Jian Zhang

**Affiliations:** 1 Institute of Immunopharmacology and Immunotherapy, School of Pharmaceutical Sciences, Shandong University, 44 Wenhua West Road, Jinan 250012, China

**Keywords:** HBV, IFN-α, Eukaryotic expression vector

## Abstract

**Background:**

Chronic hepatitis B is a primary cause of liver-related death. Interferon alpha (IFN-α) is able to inhibit the replication of hepadnavirus, and the sustained and stable expression of IFN-α at appropriate level may be beneficial to HBV clearance. With the development of molecular cloning technology, gene therapy plays a more and more important role in clinical practice. In light of the findings, an attempt to investigate the anti-HBV effects mediated by a eukaryotic expression plasmid (pSecTagB-IFN-α) in vitro was carried out.

**Methods:**

HBV positive cell line HepG2.2.15 and its parental cell HepG2 were transfected with pSecTagB-IFN-α or empty plasmid by using Lipofectamine™ 2000 reagent. The expression levels of IFN-α were determined by reverse transcriptase polymerase chain reaction (RT-PCR) and ELISA methods. The effects of pSecTagB-IFN-α on HBV mRNA, DNA and antigens were analyzed by real-time fluorescence quantitative PCR (qRT-PCR) and ELISA assays. RT-PCR, qRT-PCR and western blot were employed to investigate the influence of pSecTagB-IFN-α on IFN-α-induced signal pathway. Furthermore, through qRT-PCR and ELISA assays, the suppressive effects of endogenously expressed IFN-α and the combination with lamivudine on HBV were also examined.

**Results:**

pSecTagB-IFN-α could express efficiently in hepatoma cells, and then inhibited HBV replication, characterized by the decrease of HBV S gene (HBs) and HBV C gene (HBc) mRNA, the reduction of HBV DNA load, and the low contents of hepatitis B surface antigen (HBsAg) and hepatitis B e antigen (HBeAg). Mechanism research showed that the activation of Janus kinase (JAK)-signal transducer and activator of transcription (STAT) signal pathway, the up-regulation of IFN-α-induced antiviral effectors and double-stranded (ds) RNA sensing receptors by delivering pSecTagB-IFN-α, could be responsible for these phenomena. Furthermore, pSecTagB-IFN-α vector revealed effectively anti-HBV effect than exogenously added IFN-α. Moreover, lamivudine combined with endogenously expressed IFN-α exhibited stronger anti-HBV effect than with exogenous IFN-α.

**Conclusion:**

Our results showed that endogenously expressed IFN-α can effectively and persistently inhibit HBV replication in HBV infected cells. These observations opened a promising way to design new antiviral genetic engineering drugs based on IFN-α.

## Background

Hepatitis B virus (HBV) infection is a serious problem of public health with about 400 million HBV carriers worldwide. HBV-induced hepatocellular carcinoma (HCC) and cirrhosis have made it become a major cause of liver-related mortality, leading to one million deaths annually [1]. Immune aggression to HBV-infected cells is the main reason for liver injury and pathological progression during viral infection. The purpose of drug treatment for chronic hepatitis B (CHB) is to suppress HBV replication steadily, recover normal immune response and delay the progression of liver pathological process [2]. At present, there are two major kinds of drugs used for HBV treatment: (I) nucleoside analogues and (II) IFN-α and its derivates [3]. Nucleoside analogues (e.g., lamivudine, telbuvidine and adefovir,) directly inhibit HBV DNA synthesis and decrease viral replication. But this therapy must last for a long time, resulting in high cost. Furthermore, drug resistance will be induced in most patients during long-term application of nucleoside analogues. An increasing durability of treatment response can be achieved by IFN-α, a cytokine with antiviral and immunoregulatory functions [4].

Many studies have shown that IFN-α plays an important role in inhibiting viral replication by up-regulating many antiviral proteins, such as 2′-5′oligoadenylate synthetase (2′-5′-OAS)/endoribonuclease L (RNase L), interferon stimulated gene 56 (ISG56) family, APOBEC3 family, and Mx family proteins [5]. Cellular toll-like receptors (TLR), RIG-I-like receptors (RLR), and nucleotide organization domain-like receptors (NLR) can sense danger- and pathogen-associated RNAs. The detection of these RNAs will activate many signal pathways to induce interferon production. In turn, interferon binds its receptor will initiate signaling and lead to the up-regulation of these RNA receptors, promoting the clearance of non-self RNAs. Finally, these processes result in signaling cascade and further promote the induction of interferon to clear the invading pathogens [6]. In case an antiviral state is induced in the infected cells, viral nucleic acid or protein synthesis will be suppressed, and cell apoptosis may be initiated, and the invading HBV virus will be hardly able to replicate in hepatocytes [7]. In addition, type I IFN can activate various immune cells, including NK cells, NKT cells and DC cells [8], and up-regulate the expression of major histocompatibility complex class I(MHC-I) molecules, triggering the effective antiviral immune reactions [9]. However, variety of HBV derived protein components can interfere with the signal pathway of IFN-α by different ways [10], and part of HBV patients are unable to respond to conventional IFN-α therapy [11]. Therefore, the lasting and stable expression of IFN-α at appropriate level may enhance the local hepatic immune response and improve the curative effect of IFN-α.

Now, there are two major forms of IFN-α in the market: ordinary interferon and Peg-IFN-α [12]. The half-life of ordinary interferon is short, so patients have to accept regular intramuscular injection or subcutaneous injection. Peg-IFN-α enhances the half-life of the interferon in serum and improved its bioavailability; however, PEG reduces the specific activity of IFN-α by reducing receptor affinity and subsequent biopotency [13]. No matter what the type of IFN-α is, flu-like symptoms such as headache, muscle aches, chills and fever, will occur with each injection. These adverse reactions can range from minor to serious, and happen in more than half of patients [14]. Therefore, developing a novel IFN-α dosage form or liver-targeted drug delivery system is a new strategy to improve its curative effect.

So far, there are a lot of successful explorations about targeted delivery and in situ gene expression in the field of gene therapy [15]. Treatment of primary hepatocytes from woodchuck hepatitis virus (WHV)-infected woodchucks with an adenoviral vector encoding woodchuck specific IFN-α (wIFN-α) resulted in the suppression of WHV replication [16]. Moreover, the woodchucks remedied by intrahepatic injection of a high-capacity adenoviral vector encoding murine IL-12, showed a reduction of hepatic WHV DNA load, a decrease of surface antigen and e antigen [17]. In addition, IFN-α delivered by T-cell Receptor-Like antibodies could restrain HBV replication in hepatocytes [18]. So, we hypothesized that human IFN-α gene carried by an expressing vector might be a strategy to increase the efficacy of IFN-α-based treatment for hepatitis B.

In this study, we studied the anti-HBV function of IFN-α delivered by an expressing vector pSecTagB. We found recombinant vector pSecTagB-IFN-α could express IFN-α efficiently in hepatocytes, and this endogenously expressed IFN-α exhibited stronger anti-HBV activity than exogenously added human IFN-α.

## Results

### hIFN-α is efficiently expressed in hepatocytes transfected with recombinant pSecTagB-IFN-α vector

**Figure 1:**
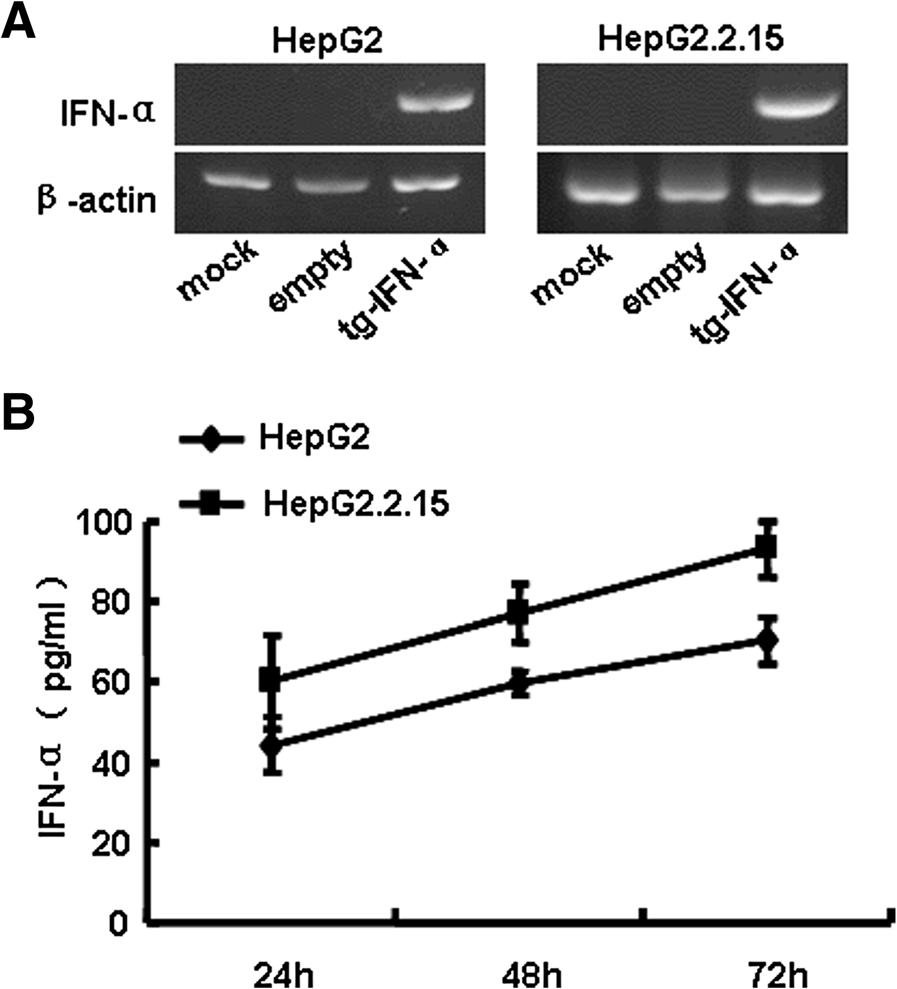
**hIFN-α was efficiently expressed in hepatocytes transfected with recombinant pSecTagB-IFN-α vector.** HepG2 and HepG2.2.15 cells were transfected with pSecTagB-IFN-α or empty plasmid at a final concentration of 1 μg/mL and 1.6 μg/mL, respectively. **(A)** After 48 h, RNAs were isolated as described in Materials and Methods, and RT-PCR analysis was performed to evaluate pSecTagB-IFN-α transfection efficiency. One representative of three independent experiments was shown. **(B)** The supernatants were harvested after 24 h, 48 h and 72 h. The level of IFN-α was measured by ELISA. Data are expressed as the mean ± SD from at least three independent experiments.

In order to investigate whether human IFN-α (hIFN-α) could be efficiently expressed in hepatocytes transfected with pSecTagB-IFN-α vector, HBV positive cell line HepG2.2.15, which chromosome was integrated by HBV genome, and its parental cell line HepG2 were treated with this recombinant vector. The data showed hIFN-α was successfully transcripted (Figure 1A) and produced (Figure 1B) in the two kinds of hepatoma cells transfected with pSecTagB-IFN-α vector. From Figure 1B we found the concentration of hIFN-α produced by HepG2 and HepG2.2.15 cells was 44 and 60 pg/mL, respectively, 24 h post of pSecTagB-IFN-α vector treatment. Cell proliferation assays demonstrated that delivering pSecTagB-IFN-α couldn’t affect the growth of hepatoma cells (Additional file 1: Figure S1). Therefore, the influence of cell number on the content of IFN-α in the supernatants and HBV load in the following experiments was excluded. These results suggested that this recombinant eukaryotic expression vector could express hIFN-α efficiently in hepatocytes.

### pSecTagB-IFN-α inhibits HBV replication

**Figure 2:**
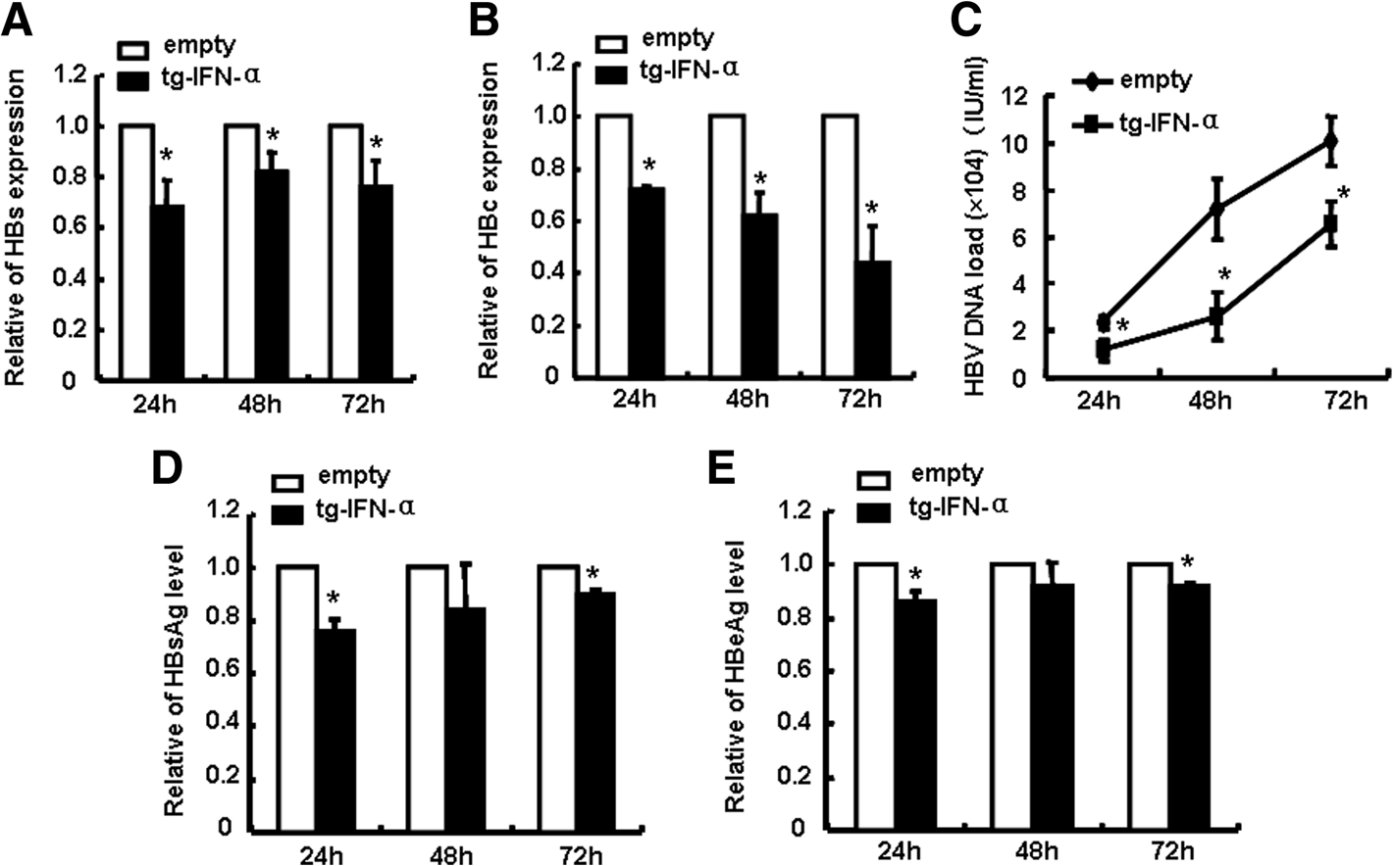
**pSecTagB-IFN-α inhibited HBV replication. (A**, **B)** HepG2.2.15 cells were transfected with pSecTagB-IFN-α or empty plasmid, and RNAs were collected after 24 h, 48 h and 72 h. The mRNA levels of HBs and HBc were measured by qRT-PCR. **(C)** HBV DNA level was quantified by fluorescence real-time PCR. **(D**, **E)** The supernatants were harvested after 24 h, 48 h and 72 h. HBsAg and HBeAg levels were assessed by ELISA. All histograms show mean values from three independent experiments and are expressed as the mean ± SD. *P < 0.05: versus empty plasmid transfected group.

Interferon remains a benchmark therapy for CHB [13]. We further investigated whether pSecTagB-IFN-α could play anti-HBV role by an autocrine hIFN-α pathway. HepG2.2.15 cells were transfected with pSecTagB-IFN-α vector or empty vector, and then HBV replication level was evaluated. As shown in Figure 2A,B the mRNA levels of HBs and HBc was decreased to 64% and 68%, respectively, 24 h post of treatment; HBV DNA load (Figure 2C) and the contents of HBsAg and HBeAg (Figure 2D,E) were also reduced significantly by pSecTagB-IFN-α transfection. These results indicated that pSecTagB-IFN-α vector could suppress HBV life activities.

### pSecTagB-IFN-α activates JAK-STAT signal pathway and promotes the induction of IFN-stimulated genes

**Figure 3:**
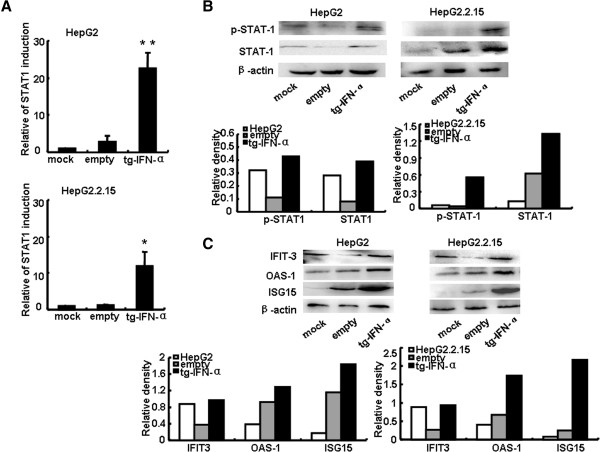
**pSecTagB-IFN-α activated JAK-STAT signal pathway and promoted the induction of IFN-stimulated genes.** The pSecTagB-IFN-α or empty plasmid was transfected into HepG2 cells and HepG2.2.15 cells at a final concentration of 1 μg/mL and 1.6 μg/mL, respectively. **(A)** After 24 h, the mRNA levels of STAT1 were measured by qRT-PCR. Data are expressed as the mean ± SD from at least three independent experiments. *P < 0.05, **P < 0.01: versus empty plasmid transfected group. **(B)** Protein levels were evaluated by western blot. One representative of three independent experiments was shown. **(C)** Western blot analysis of the protein levels of ISG15, OAS-1 and IFIT-3 in HepG2 cells and HepG2.2.15 cells were performed after transfected for 48 h. These experiments were repeated at least three times.

JAK-STAT pathway is the most crucial one in IFN-α-activated signaling pathways. STAT1 and STAT2 are important transcriptional factors of JAK-STAT pathway, and the phosphorylated STATs translocate to the nucleus and direct the transcription of hundreds of IFN-stimulated genes [19]. So, we further assessed whether transfection of pSecTagB-IFN-α vector could activate JAK-STAT pathway. By qRT-PCR and western blot analysis, we found that the levels of STAT1 and p-STAT1 were up-regulated in HepG2 cells and HepG2.2.15 cells by pSecTagB-IFN-α vector transfection (Figure 3A,B). Additionally, typical IFN-α-induced antiviral effectors, including ISG15, OAS-1 and IFIT-3, were also increased in HepG2 and HepG2.2.15 cells significantly (Figure 3C). These results suggested that JAK-STAT pathway was activated by transfection with human IFN-α expressing vector.

### Some dsRNA sensing receptors are up-regulated by IFN-α over-expression

**Figure 4:**
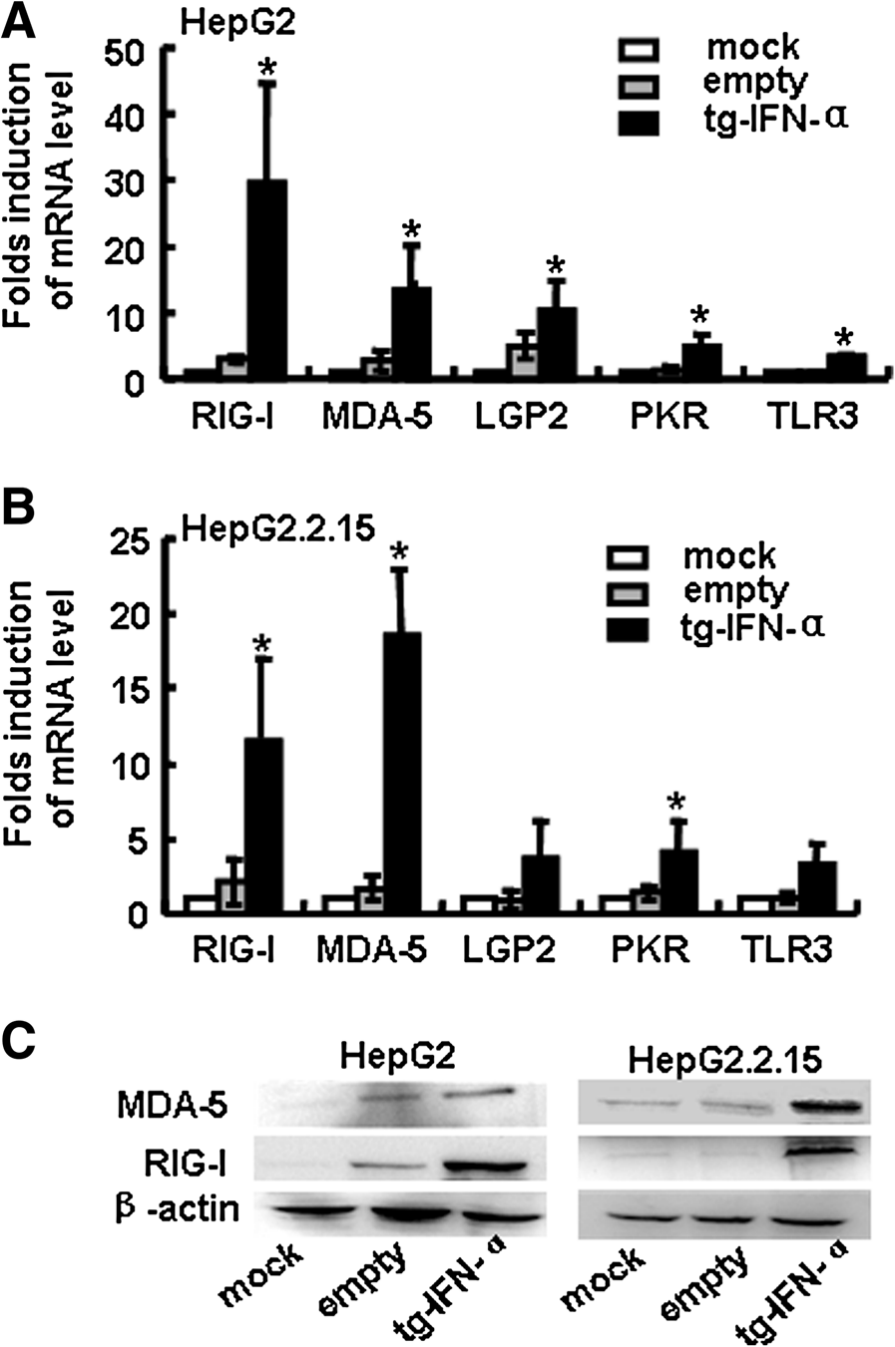
**Some dsRNA sensing receptors were up-regulated after IFN-α over-expression. (A**, **B)** HepG2 cells and HepG2.2.15 cells were transfected with pSecTagB-IFN-α or empty plasmid as previously described. Histogram showed the relative mRNA expression of RIG-I, MDA-5, LGP2, PKR and TLR3 by qRT-PCR after transfected for 48 h. Data are expressed as the mean ± SD from at least three independent experiments. * *P < 0.05*: versus empty plasmid transfected group. **(C)** Western blot analyzed the activation of RIG-I and MDA-5 by delivering pSecTagB-IFN-α into HepG2 cells and HepG2.2.15 cells after 48 h. One representative of three independent experiments was shown.

As a ligand of dsRNA receptor, double-stranded (ds) RNA is synthesized during the replication of DNA and RNA viruses. The recognition of dsRNA by its receptor leads to the production of type I IFN, which is important for the elimination of viruses [20]. Recent studies have uncovered that type I IFN can enhance the sensitivity to influenza virus by up-regulating dsRNA receptors which may contribute to the clearance of virus [21]. It has been reported that IFN-α stimulate the expression of TLR3, PKR, RIG-I and MDA-5 in keratinocytes [22]. We therefore assumed whether the expression of dsRNA receptors in hepatocytes would be increased by transfecting human IFN-α expressing vector. As expected, the mRNA levels (Figure 4A,B) of RIG-I, MDA-5, LGP2, PKR and TLR3, especially RIG-I and MDA-5, were increased in HepG2 and HepG2.2.15 cells by the transfection of pSecTagB-IFN-α vector, as well as the protein levels of RIG-I and MDA-5 (Figure 4C). These data suggested that some dsRNA sensing receptors were up-regulated by over-expression of IFN-α.

### Endogenously expressed IFN-α is more effective in inhibiting HBV than exogenously supplemented IFN-α

**Figure 5:**
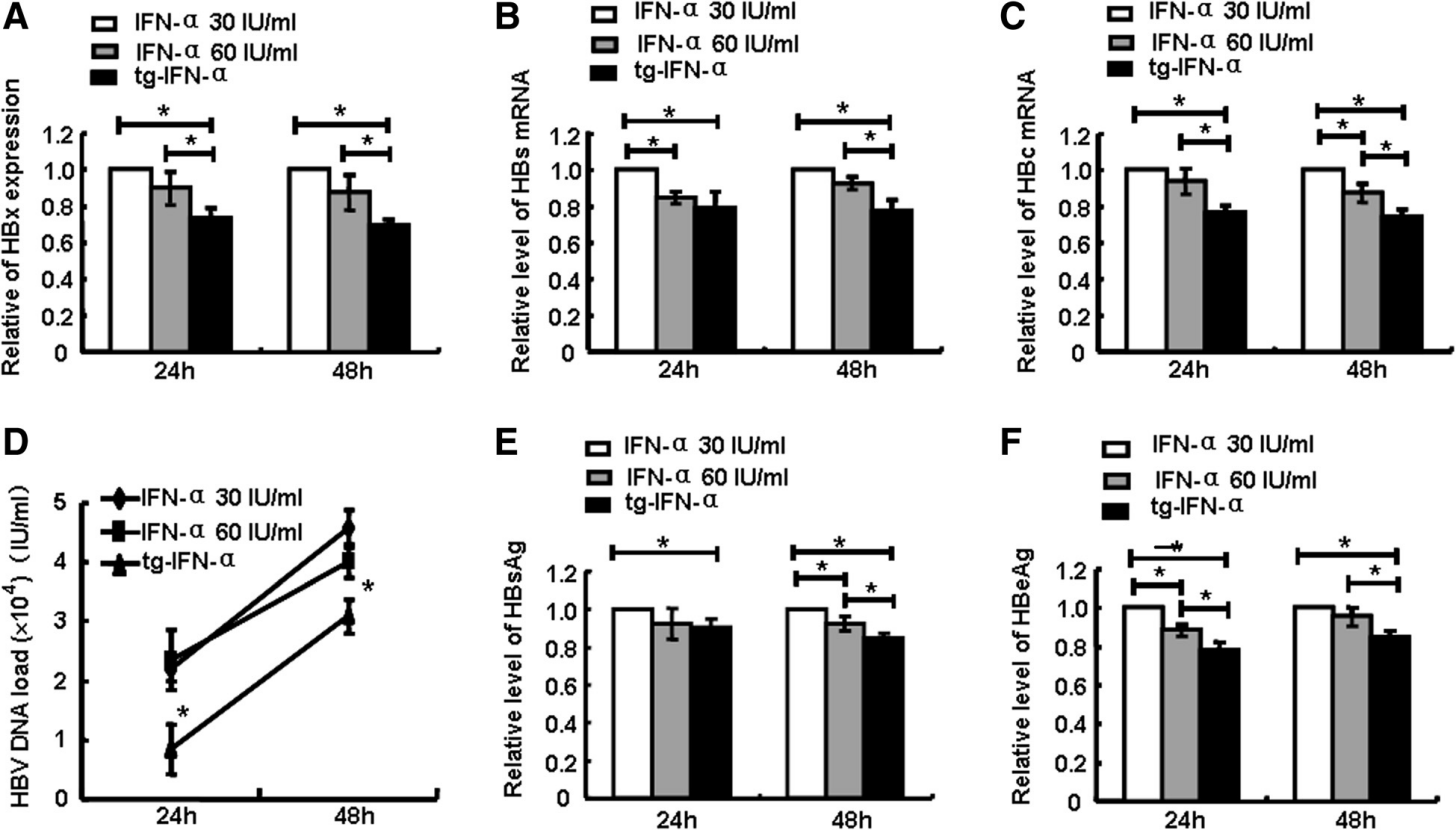
**Endogenously expressed IFN-α was more effective in inhibiting HBV than exogenous IFN-α. (A**-**C)** HepG2.2.15 cells were transfected with pSecTagB-IFN-α or treated with IFN-α2a at the dose of 30 IU/ml or 60 IU/ml,and then RNAs were isolated after 24 h and 48 h. The mRNA levels of HBx, HBs and HBc were quantified. **(D)** HBV DNA load was quantified by fluorescence real-time PCR. **(E**, **F)** HBsAg and HBeAg levels were tested by ELISA. Data represented of three independent experiments and are expressed as the mean ± SD. *P < 0.05: versus IFN-α2a stimulated group.

To compare the anti-HBV effects between endogenously expressed IFN-α and exogenously added IFN-α, HepG2.2.15 cells were transfected with pSecTagB-IFN-α vector, or treated with recombinant hIFN-α at the dose of 30 IU/mL (~80 pg/mL) or 60 IU/mL, which was similar or twice the content of hIFN-α in the supernatant from HepG2.2.15 cells transfected with pSecTagB-IFN-α vector for 48 h. Then, HBV replication ability was assessed. The results showed the exogenous hIFN-α didn’t show significant inhibitory role in HBV (Additional file 2: Figure S2); however, endogenously expressed hIFN-α display stronger anti-HBV effect than exogenous hIFN-α which was even at the dose of 60 IU/mL, accompanied with the down-regulation of mRNA levels of HBx, HBs and HBc (Figure 5A-C) and HBV DNA load (Figure 5D), as well as the contents of HBsAg and HBeAg (Figure 5E,F). These data showed that transfection of IFN-α plasmid could be more efficient in suppressing HBV than exogenous recombinant hIFN-α.

### Lamivudine combined with pSecTagB-IFN-α displays enhanced anti-HBV effect than with exogenously supplemented IFN-α

**Figure 6:**
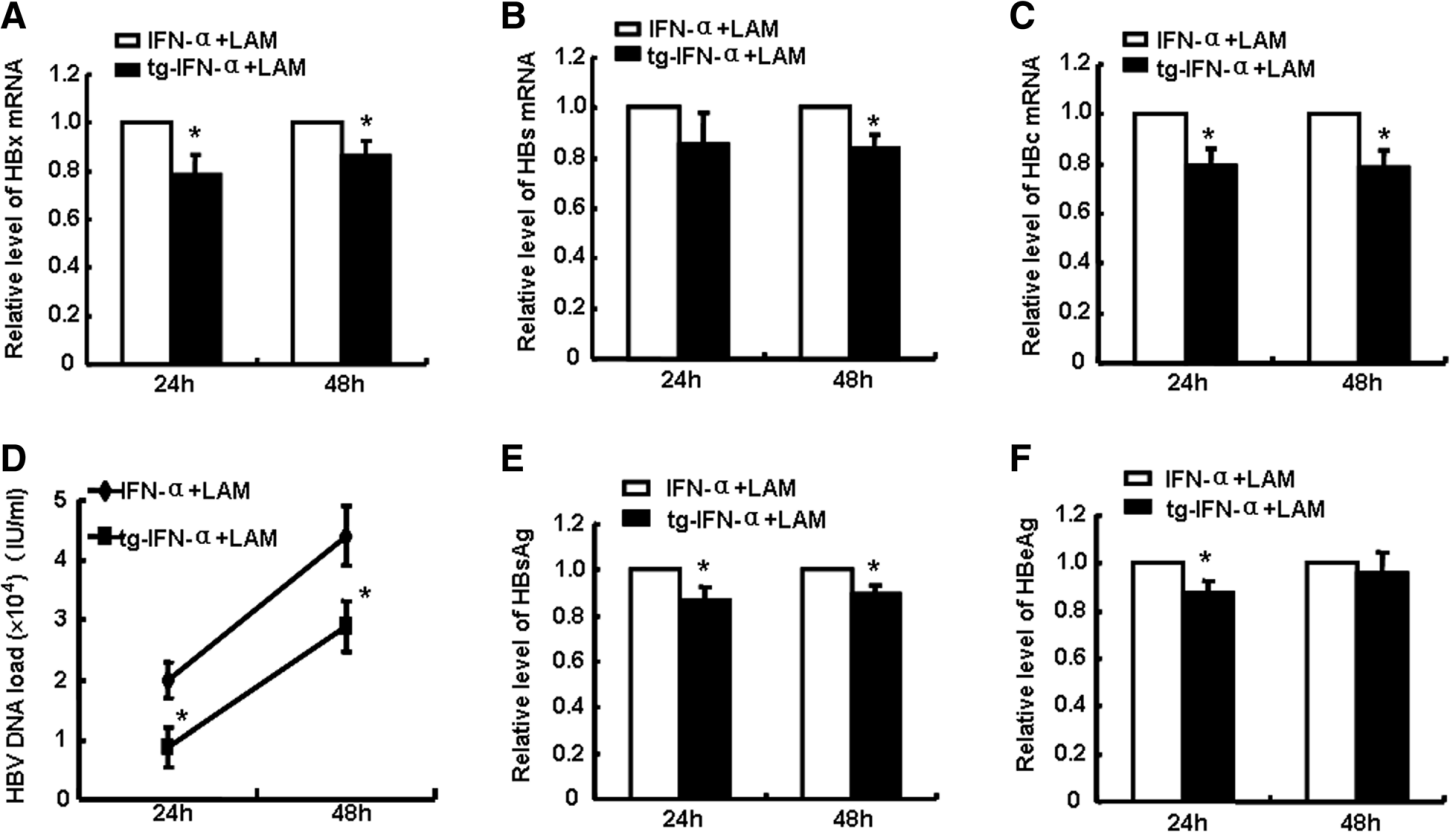
**Lamivudine combined with pSecTagB-IFN-α displayed enhanced anti-HBV effect than with exogenously supplemented IFN-α. (A**-**C)** HepG2.2.15 cells were transfected with pSecTagB-IFN-α and treated with lamivudine (3 μmol/L), or treated with IFN-α2a (30 IU/mL) and lamivudine (3 μmol/L). Then, RNAs were extracted after 24 h and 48 h. The relative mRNA expression of HBx, HBs and HBc were examined by qRT-PCR. **(D)** The level of HBV DNA was quantified by fluorescence real-time PCR. **(E**, **F)** The contents of HBsAg and HBeAg were detected by ELISA. Data are expressed as the mean ± SD from at least three independent experiments. *p < 0.05: versus the combination of exogenous IFN-α2a and lamivudine stimulated group.

Lamivudine, a nucleoside analogue available for HBV treatment, is restricted by the high incidence of resistance [23]. It is noteworthy that the combination of lamivudine and IFN-α can induce a more meaningful antiviral effect than lamivudine monotherapy [24]. Subsequently, we further examined whether the combination of endogenously expressed hIFN-α and lamivudine could play augmented anti-HBV effect. As shown in Figure 6, HepG2.2.15 cells were transfected with pSecTagB-IFN-α and treated with lamivudine simultaneously, or treated with IFN-α2a (30 IU/ml) combined with lamivudine. The results showed that lamivudine combined with pSecTagB-IFN-α transfection would display a stronger anti-HBV effect at a certain degree than with exogenous hIFN-α (Additional file 3: Figure S3), which was characterized by a reduction of HBx, HBs and HBc mRNA (Figure 6A-C), and lower level of HBV DNA load (Figure 6D), and a decline in HBsAg and HBeAg (Figure 6E,F). These findings suggested that the combination of endogenous hIFN-α and lamivudine might be beneficial for anti- HBV treatment.

## Discussion

To date, IFN-α is widely used in clinical therapy for patients with chronic hepatitis B [25]. However, there are many limitations attenuating the curative effect of IFN-α, such as short half-life, serious side effects and instable plasma drug concentration [26]. It is necessary to develop new strategies to overcome these restrictions. In recent years, there are many significant developments about the concept of taking gene therapy as an approach in the treatment of disorders [27], and many advantages in the field of gene therapy, such as targeted drug delivery and continuous infusion, have made it possible that gene therapy play an increasing important role in clinical application [28]. In the present study, in an attempt to get better therapeutic effect for HBV, a recombinant eukaryotic expression vector encoding human IFN-α was used.

The experimental results demonstrated that human hepatocytes treated with human IFN-α expressing vector pSecTagB-IFN-α resulted in the secretion of IFN-α (Figure 1), concomitant with the decreased HBV replication (Figure 2). Mechanism research showed the induction of typical antiviral proteins (Figure 3), and the up-regulation of some dsRNA sensing receptors (Figure 4) could partly account for these phenomena. Furthermore, we compared the anti-HBV effect of pSecTagB-IFN-α with exogenously added IFN-α, and the results indicated that pSecTagB-IFN-α exhibited stronger anti-HBV effect than exogenously added hIFN-α at the dose of 60 IU/mL, which was two times of the content of IFN-α produced by pSecTagB-IFN-α- transfected HepG2.2.15 cells (Figure 5).

Lamivudine, the first oral nucleotide analogue approved for CHB patients, is beneficial to HBeAg seroconversion, normalization of ALT, inhibition of HBV replication and slowdown of fibrosis [29]. In spite of high rate of drug resistance, so far, lamivudine is the most widely used nucleoside analogues for HBV treatment owing to its low price, safety and effectiveness [30]. Many previous studies reported that a combination of IFN-α and lamivudine achieved some therapeutic effects [31,32]. Here, in comparison to the treatment with exogenously added hIFN-α combined with lamivudine, the combination of endogenously expressed hIFN-α and lamivudine could restrain HBV more effectively at a certain degree (Figure 6). In addition, our study showed the elevated levels of MHC I and Fas in HCC cells transfected with pSecTagB-IFN-α (Additional file 4: Figure S4), which might enhance T cell-mediated immune responses.

In view of these, we speculate that the autocrine pathway of human IFN-α in hepatocytes may be a more efficacious therapeutic method for HBV treatment than exogenous hIFN-α. Through liver cell specific expression vector which contain hepatocyte specific α-fetoprotein (AFP) promoter [33], endogenous expression of IFN-α may display therapeutic value in the treatment of CHB, inhibiting the replication of HBV and resulting in a reduction of hepatic inflammation, slowing the process of chronic liver injury. At the same time, it provides a new approach for HBV treatment based on the combination of gene therapy and the traditional drug therapy.

## Conclusion

Endogenous expression of human IFN-α possess the feature to inhibit HBV more efficaciously and enduringly. These observations might have therapeutic value in the treatment of chronic HBV infection.

## Materials and methods

### Cell line and plasmids

Human hepatoma cell lines HepG2 and HBV-positive HepG2.2.15 cells conserved in our laboratory were cultured in DMEM (GIBCO/BRL, Grand Island, N.Y. USA) containing 10% fetal bovine serum in a humidified incubator with 5% CO_2_ at 37°C. The empty expression vector pSecTagB was obtained from Invitrogen (CA, USA). The recombinant vector (pSecTagB-IFN-α) which contains IFN-α1 cDNA was constructed in our laboratory.

### Transfection

HepG2 or HepG2.2.15 cells were seed at a density of 2.0 × 10^5^ cells/mL, and then transfected with pSecTagB-IFN-α or empty plasmid using Lipofectamine™ 2000 (Invitrogen, CA, USA) according to the manufacturer’s instruction. The final concentration of plasmid was 1 mg/mL in HepG2 cells and 1.6 mg/mL in HepG2.2.15 cells.

### Reverse transcriptase polymerase chain reaction (RT-PCR) assay

Total RNA was extracted by Trizol Reagent (Invitrogen, Carlsbad, CA, USA) according to the manufacturer’s protocol. The concentration and quality of the extracted RNA were determined by measuring light absorbance at 260 nm (A260) and a ratio of (A260/A280). cDNA was synthesized from 3 μg RNA using M-MLV reverse transcriptase (Promega, Madison, USA), and PCR was performed in a total volume of 25 μL as depicted previously [34]. DNA sequences of primers (forward primer; reverse primer) were β–actin (GTGGGGCGCCCCAGGCACCA, CTCCTTAATGTCACGCACGA), IFN-α (TTAGGATCCATGGCCTCGCCCTTT, CGCGAATTCGTTATTCCTTCCTCC).

### Western blot

Cells were harvested 24 h or 48 h after transfecteion of pSecTagB-IFN-α or empty plasmid. Then, proteins were extracted by Total Protein Extraction Kit (BestBio Shanghai, China). Western blot analysis was performed as previously described [35]. Antibodies for ISG15, STAT-1, *p*-STAT1, MDA-5 and RIG-I were obtained from Cell Signaling Technology (NewEngland BioLabs Inc.); and antibody for β-actin, OAS-1 and IFIT-3 were purchased from Santa Cruz Biotechnology (Santa Cruz, CA, USA).

### Real-time PCR

Real-time fluorescence quantitative PCR (qRT-PCR) was performed using the SYBR Green Realtime PCR Master Mix (Code No. QPK-201, Toyobo, Japan). Glyceraldehyde 3-phosphate dehydrogenase (GAPDH) was used as an internal standard for the quantification of the PCR. The primers were synthesized by the Beijing Genomics Institute (Beijing, China) and listed in Table 1.

**Table 1:** Primers used for real-time PCR

**Gene**	**Sequence (5′– > 3′)**	**Size (bp)**
GAPDH	F:CATGGGTGGAATCATATTGGAA	155
	R:GAAGGTGAAGGTCGGAGT	
HBV-X	F:CCGTCTGTGCCTTCTCATCTGC	256
	R:ACCAATTTATGCCTACAGCCTCC	
HBV-S	F:ATCCTGCTGCTATGCCTCATCTT	314
	R:ACAGTGGGGGAAAGCCCTACGAA	
HBV-C	F:CTGGGTGGGTGTTAATTTGG	186
	R:TAAGCTGGAGGAGTGCGAAT	
PKR	F:ATGATGGAAAGCGAACAAGG	76
	R:TTCTCTGGGCTTTTCTTCCA	
RIG-I	F:CTTCACATGGATTCCCCAG	187
	R:GGCATGTTACACAGCTGACG	
MDA-5	F:GGAGTTTTCAAGGATTTGAGC	479
	R:AGTTTGGCAGAAGGAAGTGTC	
LGP2	F:CCACAGCCACCATGCAGTTGAT	242
	R:CAACTGAAGGTAGCCGGGA	
TLR3	F:GACCTCTCCATTCCTGGC	156
	R:TCACTTGCTCATTCTCCCTT	

### Analysis of HBV DNA

Viral particles in the supernatants were quantified by fluorescence real-time PCR according to the instructions of the kit for Quantification of HBV DNA (Da-An, Guangzhou, China).

### ELISA

Supernatants were harvested from HCC cells after transfected with pSecTagB-IFN-α or empty plasmid, and the amounts of HBeAg and HBsAg were detected by ELISA kit from RongSheng Biotechnology (Shanghai, China). The level of IFN-α was quantified using ELISA kits from XiTang Biotechnology (Shanghai, China). Assays were performed according to the manufacturer’s instruction.

### Cell proliferation assay

All cellular growth assays were performed in 96-well plates. HepG2 cells were seed at a density of 5 × 10^3^ cells/well and HepG2.2.15 cells were seed at a density of 1 × 10^4^ cells/well, followed by transfection of pSecTagB-IFN-α or empty plasmid using Lipofectamine™ 2000 according to the manufacturer’s description. The final concentration of plasmid was 1 mg/mL in HepG2 cells and 1.6 mg/mL in HepG2.2.15 cells, and then incubated for indicated time. Next, 15 μL MTT (10 mg/mL, Sigma, St Louis, MO, USA) solution was added, and the plates were incubated for a further 4 h at 37°C. After centrifugation, MTT solution was then eliminated, and 200 μL dimethyl-sulfoxide (DMSO; Sigma, St Louis, MO, USA) was added to each well to dissolve the formazan crystals. The absorbance at 570 nm (A570) in each well was confirmed by a scanning multi-well spectrophotometer.

### Flow cytometry

Immunofluorescence staining was performed using standard protocols. HCC cells were transfected with pSecTagB-IFN-α or empty plasmid for 48 h. The expression of cell surface MHC I and Fas were determined by flow cytometry. Briefly, the cells were washed twice in washing buffer (PBS) and resuspended in 200 μL PBS before incubation with PE-conjugated anti-HLA-abc (BD Pharmingen, USA) and PEcy5-conjugated anti-Fas (BD Pharmingen, USA) for 40 min at 4°C. Results were analyzed by FACS Calibur™ and CELL Quest™ software (Becton Dickinson, Mountain View, CA, USA).

### Statistical analyzes

All data are expressed as mean ± SD and accompanied by at least three distinct experiments. Statistical analysis was determined by a paired Student’s t-test and *P < 0.05, **P < 0.01 were considered statistically significant.

## Abbreviations

HBV: Hepatitis B virus; IFN-α: Interferon alpha; hIFN-α: Human interferon alpha; HBx: HBV X gene; HBs: HBV S gene; HBc: HBV C gene; HBsAg: Hepatitis B surface antigen; HBeAg: Hepatitis B e antigen; OAS-1: 2′-5′-oligoadenylate synthetase-1; ISG15: Interferon stimulated gene 15; IFIT-3: Interferon-induced protein with tetratricopeptide repeats-3; RIG-I: Retionic-acid inducible gene-1; MDA-5: Melanoma differentiation-associated gene-5; LGP2: Laboratory of genetics and physiology 2; TLR3: Toll-like receptor 3; PKR: Protein kinase R; ELISA: Enzyme-linked immunosorbent assay; MHC-I: Major hiatocompatibility complex class I; AFP: α-fetoprotein; GAPDH: Glyceraldehyde 3-phosphate dehydrogenase.

## Competing interest

The authors declare that they have no competing interests.

## Authors’ contributions

HY carried out most of the experiments and wrote the manuscript. ZH participated in project design and revised the manuscript. QH and CZ provided useful advices for the project. JZ is the project leader and was involved in project conception, design, data analysis, and finalization of the manuscript. All authors read and approved the final manuscript.

## Supplementary Material

Additional file 1: Figure S1 Effect of pSecTagB-IFN-α on the proliferation of hepatocytes. HepG2 cells and HepG2.2.15 cells were transfected with pSecTagB-IFN-α as described in Materials and Methods. The growth of these HepG2 and HepG2.2.15 cells were tested by MTT assay. These experiments were repeated at least three times. Click here for file

Additional file 2: Figure S2 Exogenous IFN-α couldn’t evidently play a role in suppressing HBV. HepG2.2.15 cells were stimulated with IFN-α2a at a dose of 30 IU/mL or 60 IU/mL, respectively. Then, RNAs were collected after 24 h and 48 h. The mRNA levels of HBx, HBs and HBc were quantified by qRT-PCR. Data represented of three independent experiments and are expressed as the mean ± SD. *p < 0.05: versus HepG2.2.15 or IFN-α2a stimulated group. Click here for file

Additional file 3: Figure S3 Exogenous IFN-α combined with lamivudine didn’t exhibit obviously increased anti-HBV function. HepG2.2.15 cells were stimulated with lamivudine (3 μmol/L) and IFN-α2a (30 IU/mL) simultaneously. Then, RNAs were isolated after 24 h and 48 h. The relative mRNA levels of HBx, HBs and HBc were examined by qRT-PCR. Data are expressed as the mean ± SD from three independent experiments. *p < 0.05: versus HepG2.2.15 or lamivudine stimulated group. Click here for file

Additional file 4: Figure S4 pSecTagB-IFN-α up-regulated the expression of MHC I and Fas. HepG2 cells and HepG2.2.15 cells were transfected with pSecTagB-IFN-α as previously described. After 48 h, the expressions of MHC I and Fas were tested by flow cytometry analysis. One representative of three independent experiments was shown. Click here for file
