# Supplementary figures and images for: The anti-HBV effect mediated by a novel recombinant eukaryotic expression vector for IFN-α
Source: Virol J. 2013 Aug 29;10:270. doi: 10.1186/1743-422X-10-270 (PMC3766191; doi:10.1186/1743-422X-10-270)

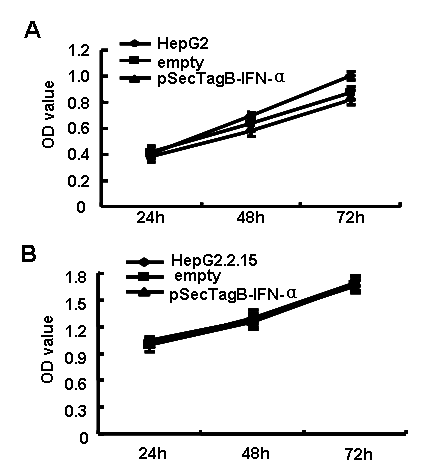

Supplement: Additional file 1: Figure S1 — Effect of pSecTagB-IFN-α on the proliferation of hepatocytes. HepG2 cells and HepG2.2.15 cells were transfected with pSecTagB-IFN-α as described in Materials and Methods. The growth of these HepG2 and HepG2.2.15 cells were tested by MTT assay. These experiments were repeated at least three times. [file 1743-422X-10-270-S1.tiff]

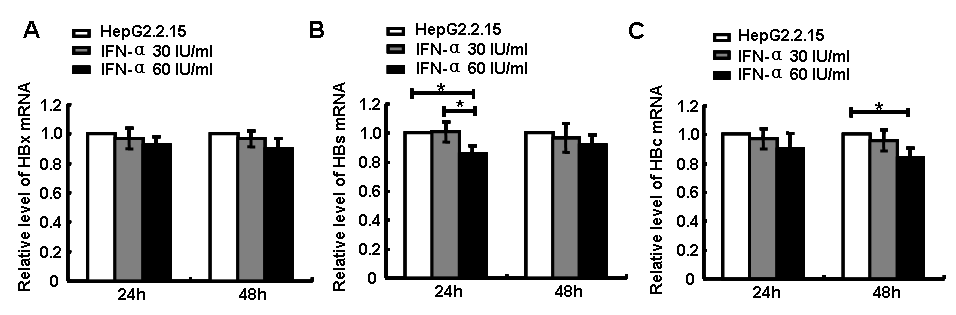

Supplement: Additional file 2: Figure S2 — Exogenous IFN-α couldn’t evidently play a role in suppressing HBV. HepG2.2.15 cells were stimulated with IFN-α2a at a dose of 30 IU/mL or 60 IU/mL, respectively. Then, RNAs were collected after 24 h and 48 h. The mRNA levels of HBx, HBs and HBc were quantified by qRT-PCR. Data represented of three independent experiments and are expressed as the mean ± SD. *p < 0.05: versus HepG2.2.15 or IFN-α2a stimulated group. [file 1743-422X-10-270-S2.tiff]

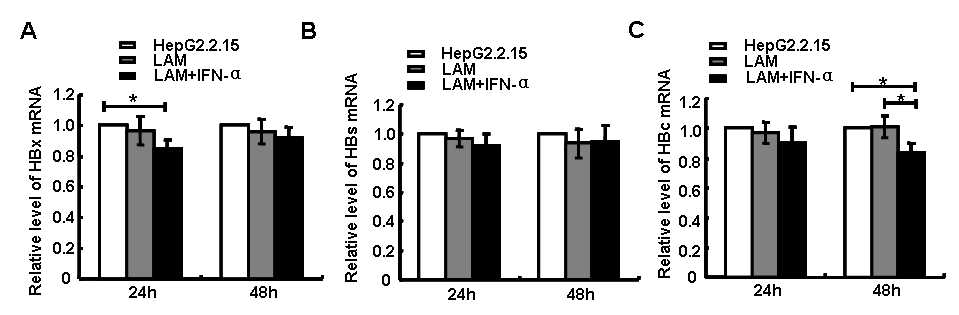

Supplement: Additional file 3: Figure S3 — Exogenous IFN-α combined with lamivudine didn’t exhibit obviously increased anti-HBV function. HepG2.2.15 cells were stimulated with lamivudine (3 μmol/L) and IFN-α2a (30 IU/mL) simultaneously. Then, RNAs were isolated after 24 h and 48 h. The relative mRNA levels of HBx, HBs and HBc were examined by qRT-PCR. Data are expressed as the mean ± SD from three independent experiments. *p < 0.05: versus HepG2.2.15 or lamivudine stimulated group. [file 1743-422X-10-270-S3.tiff]

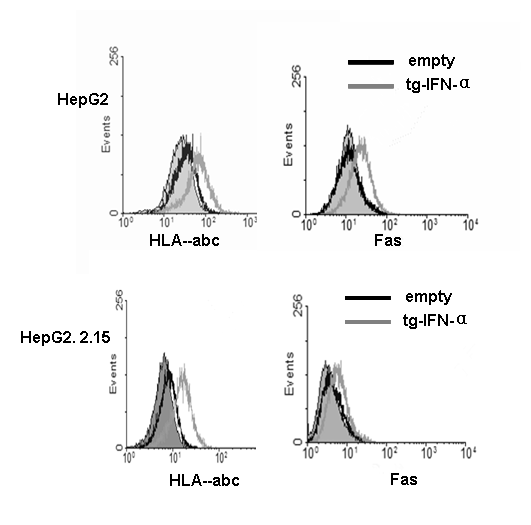

Supplement: Additional file 4: Figure S4 — pSecTagB-IFN-α up-regulated the expression of MHC I and Fas. HepG2 cells and HepG2.2.15 cells were transfected with pSecTagB-IFN-α as previously described. After 48 h, the expressions of MHC I and Fas were tested by flow cytometry analysis. One representative of three independent experiments was shown. [file 1743-422X-10-270-S4.tiff]
